# Does the public know what researchers know? Perceived task difficulty impacts adults’ intuitions about children’s early word learning

**DOI:** 10.1186/s41235-023-00493-y

**Published:** 2023-07-24

**Authors:** Melina L. Knabe, Christina C. Schonberg, Haley A. Vlach

**Affiliations:** 1 Department of Educational Psychology, University of Wisconsin-Madison, 1025 W. Johnson St., Madison, WI 53703 USA; 2 IXL, 777 Mariners Island Blvd., Suite 600, San Mateo, CA 94404 USA

**Keywords:** Language development, Word learning, Adult perceptions

## Abstract

The present study examined adults’ understanding of children’s early word learning. Undergraduates, non-parents, parents, and Speech-Language Pathologists (*N* = 535, 74% female, 56% White) completed a survey with 11 word learning principles from the perspective of a preschooler. Questions tested key principles from early word learning research. For each question, participants were prompted to select an answer based on the perspective of a preschooler. Adults demonstrated aligned intuitions for all principles except those derived from domain-general theories, regardless of experience with language development (Experiment 1). Experiment 2 revealed that perceived difficulty of a task for a preschooler impacted adults’ reasoning about word learning processes. Experiment 3 ruled out level of confidence and interest as mechanisms to explain the results. These results highlight disconnects in knowledge between the cognitive development research community and the general public. Therefore, efforts must be made to communicate scientific findings to the broader non-academic community, emphasizing children’s ability to excel at word learning in the face of task difficulty.

**Supplementary Information:**

The online version contains supplementary material available at 10.1186/s41235-023-00493-y.

## Introduction

Word learning is a central task of early childhood and serves as the building block for subsequent language development. To understand word learning, researchers have identified how children segment words in a speech stream (e.g., Saffran et al., 1996), map words to objects in the environment (e.g., Carey, 2010), and retain these word mappings across time (e.g., Vlach & Sandhofer, 2012a). Although language development research has led to great strides in our understanding of how children learn language, we do not know whether adults—as co-constructors of children’s learning—have intuitions that align with these findings. Therefore, the present study sought to understand adults’ intuitions about the science of children’s early word mapping and learning.

### How do children learn words?

A central question within word learning research is how children learn words in a world rife with *referential ambiguity* (Quine, 1960); that is, how do children learn words when it is uncertain what referent—or object—a particular word is referencing? Imagine a child hears the following sentence at the playground: “Look at the dog!”. The word “dog” could refer to the whole animal, a part of the animal, the action of the animal, or an entirely different object in the environment. After the child accurately maps the word “dog” to the correct referent, how do they then encode, retain, and retrieve this mapping across time?

Three major word learning theories have been developed to explain how children overcome referential ambiguity and learn words: constraints, sociopragmatic, and domain-general theories (e.g., Hollich et al., 2000; Saffran & Thiessen, 2007). These theories differ in their emphasis on the contribution of the learner and the environment to acquiring words, and have led to a corpus of well-studied and replicable research findings (see Table 1). It is important to note that the theories underlying these principles are not mutually exclusive; they provide different levels of explanation and children might weight these mechanisms more/less across developmental time. Constraints theories claim that children use a set of internal rules or biases to map words to objects (e.g., Markman, 1989; Woodward & Markman, 1998). For instance, the *whole object assumption* describes children’s tendency to assume a novel word refers to a whole object rather than to its parts/properties (Markman & Wachtel, 1988). This principle is considered evidence for constraints theories of word learning because children display a bias that is not learned.

**Table 1:** Principles derived from major word learning theories

Task	Theory	Description	Example Reference(s)
Mutual exclusivity assumption	Constraints theory	A speaker provides novel labels for several familiar and novel objects. Participants tend to apply novel labels to the novel object as opposed to the familiar object	Markman (1989), Merriman and Bowman (1989)
Whole object assumption	Constraints theory	A speaker labels novel objects with various unfamiliar parts. Participants tend to apply novel labels to the whole object as opposed to a part of the object	Markman & Wachtel, (1988)
Taxonomic bias	Constraints theory	A speaker presents participants with a target object (e.g., dog), followed by thematic associate (e.g., bone) and a taxonomic associate (e.g., another dog). The target object is labeled with a novel word (e.g., “wug”). When asked to find another “wug”, participants are more likely to choose the taxonomic associate	Markman and Hutchinson (1984)
Shape bias	Constraints theory	A speaker labels a novel object (e.g., “wug”). Participants are then shown three other objects that match in size, color, or shape. When asked to find another “wug”, participants are more likely to choose the object that matches in shape	Diesendruck and Bloom (2003), Landau et al. (1988)
Pointing during learning	Sociopragmatic theory	A speaker labels a novel object while pointing at or not pointing at the novel object. Participants are more likely to learn the label of the novel object when the speaker is pointing at the object	Booth et al. (2008)
Looking to visible object	Sociopragmatic theory	A speaker labels a novel object while looking at or not looking at the novel object. Participants are more likely to learn the label of the novel object when the speaker is looking at the object	Booth et al. (2008)
Looking to non-visible object	Sociopragmatic theory	A speaker plays with a novel object and places it in one of two buckets in front of the participant. The novel object is no longer visible to the participant. The speaker labels the object while it is in the bucket. Then, the experimenter removes the labeled object from the bucket and another object in the second bucket that was not labeled. Participants are able to accurately identify the novel object that labelled while not visible	Baldwin (1995)
Overheard speech	Sociopragmatic theory	Two speakers label a series of novel objects in the presence of the participant. The labeling events are not explicitly directed at the participant. Nonetheless, the participant can accurately map the labels to the objects	Tomasello et al. (1995)
Massed vs. spaced learning	Domain-general theory	A speaker provides novel objects and labels on a massed (i.e., in immediate succession) or spaced (i.e., distributed across time) schedule. Participants display higher retention of novel objects presented on a spaced schedule	Vlach et al. (2008), Vlach & Sandhofer (2012b)
Cross-situational word learning	Domain-general theory	A speaker provides several novel objects and labels on-screen in a single trial (“This is a *wug.* This is a *dax.*”). At first, it is ambiguous which word corresponds to which object. Across the learning phase, however, words and objects co-occur in a reliable manner. Participants can accurately map words to objects presented cross-situationally	Smith and Yu, (2008), Vlach and DeBrock, (2017)
)Same vs. varied context	Domain-general theory	A speaker provides novel objects and labels on a consistent background (e.g., same patterned cloth) versus a varied background (e.g., different patterned cloths). Participants display higher retention of novel objects presented on a varied background than the same background	Smith and Rothkopf (1984), Smith et al. (1978), Vlach and Sandhofer (2012a)

In contrast, domain-general theories attribute word learning to general cognitive processes of perception, attention, and memory (e.g., Samuelson & Smith, 1998; Vlach & Sandhofer, 2012a; Vlach, 2014, 2019). An example of a principle derived from domain-general theories is *massed vs. spaced learning,* which refers to the way information is distributed during learning. Children benefit from learning new words on a spaced schedule because spacing out information leads to forgetting and effortful retrieval, which boosts long-term retention (Vlach & Sandhofer, 2012a; Vlach et al., 2008).

Finally, rather than relying on learner characteristics, sociopragmatic theories posit that children primarily rely on social information, such as sociopragmatic cues (e.g., pointing, eye-gaze). Children thus acquire words by making inferences about the referential intent of speakers in their environment (e.g., Baldwin, 1995; Baldwin & Tomasello, 1998; Tomasello & Akhtar, 1995). An example of a principle derived from sociopragmatic theories is learning from *looking to a visible object.* Studies have found that children are better able to map words to objects when adults look to the object during labeling, highlighting the role of sociopragmatic cues in language learning (Booth et al., 2008).

In brief, researchers have identified several robust learning mechanisms and heuristics underlying children’s word mapping and learning, which have been observed outside of the laboratory (e.g., Cartmill et al., 2010; Leonard et al., 2021, 2022; Shneidman & Goldin-Meadow, 2012; Sobel et al., 2011).

### How do adults think children learn language?

Although researchers have spent considerable time studying children’s word learning, little time has been devoted to examining how the public thinks about these phenomena. This is a critical gap to address because adults serve as the architects and co-constructors of children’s learning environments (MacPhee, 1983; Miller et al., 1988; Rodrigo & Triana, 1996). That is, in addition to children generating their own learning opportunities, the opportunities that adults provide can shape children’s language development (e.g., Mahr & Edwards, 2018; Montag et al., 2018; Rowe, 2012; Smith et al., 2011, 2018; Tamis-LeMonda et al., 2018; Weisleder & Fernald, 2013). If adults demonstrate correct notions of how children learn—that is, alignment between their intuitions and established theories of word learning—there may be no need to intervene. However, if adults possess incorrect notions of how children learn, language development might not be supported effectively. A first step is thus to understand whether adults’ intuitions in fact align with research findings. That is, does the public know what researchers know about children’s word learning? Knowing whether adults’ intuitions do or do not align with research findings allows researchers to target gaps in knowledge. Once these gaps are identified, the next step is to determine whether adults’ beliefs shape their behavior, and whether their behavior shapes children’s language outcomes.

What intuitions might adults hold about children’s behavior in word learning situations? One hypothesis is that adults hold intuitions about children’s language learning that *align* with research findings. These aligned intuitions may arise from adults’ conceptions about their own learning, as well as their informal experience with children’s language development. Through informal experience, advice from health care professionals, and educational campaigns, adults might have learned which strategies benefit language learning (e.g., Golinkoff et al., 2019; Greenwood et al., 2017). For instance, adults likely intuit that pointing at an object while repeating its name supports language learning. They might also recognize that picking up a whole object and providing its label allows children to more effectively map the word to the object. Together, these experiences shape adults’ reasoning about children’s language development.

An alternative hypothesis is that adults hold intuitions that *do not align* with key research findings. These misaligned intuitions may arise from adults’ general misconceptions about learning. For example, prior research has shown that adults have inaccurate perceptions of domain-general principles in human memory, such as spaced learning (Kornell & Bjork, 2008). When asked how well they learned information presented on a massed versus spaced schedule, adults display a *massed bias*; they claim that massing information is more beneficial than spacing information (e.g., Kornell & Bjork, 2008; McCabe, 2011; Zechmeister & Shaughnessy, 1980). Spaced learning falls under the *desirable difficulties* framework, which suggests that more effortful learning conditions enhance long-term retention (Bjork, 1994; Bjork & Bjork, 2009; Dempster, 1988; Roediger & Karpicke, 2006). Many adults do not appreciate the efficacy of introducing deliberate difficulties during learning; even though spaced learning is more effective than massed learning, it feels more difficult and ineffective (Ariel & Karpicke, 2018; Hui et al., 2021). Therefore, adults might hold misaligned intuitions about how children learn new information, such as words.

A potential contributing factor to the alignment between adults’ intuitions and research is their experience with children’s language development. That is, adults with more experience or expertise with children’s language development may have intuitions more aligned with key research findings (Chi et al., 2014; Hoffman, 1998). In the context of word learning, parents and Speech Language Pathologists (SLPs) would be considered experts with children’s language learning and instruction. Parents have *informal* experience with the trajectory of children’s word learning: They observe their own children’s and their children’s peers’ language learning. Moreover, language milestones are discussed in pediatric visits and parents are aware of their child’s growing ability to communicate. SLPs have *formal* experience with the trajectory of children’s word learning through their clinical training and practice. Although SLPs work with various disorders and demographic groups, they are considered experts in communication sciences more broadly.

Based on the aforementioned research, we hypothesized that adults would have intuitions that aligned with research findings, except for principles derived from domain-general theories. After all, domain-general theories rely on an understanding of how memory operates and learning is strengthened; a common source of misalignment for adults. Furthermore, we hypothesized that parents and SLPs would have intuitions that were more aligned with research findings for all principles. The current studies tested these predictions and isolated a potential mechanism to explain adults’ intuitions about word learning.

### Current study

The goal of the current research was to assess adults’ intuitions about children’s word learning; a first step in understanding the link between adults’ beliefs and behavior, and children’s learning outcomes. Experiment 1 examined whether nonexpert (undergraduate students, general public) and expert (parents, SLPs) adults’ intuitions aligned with key research findings in language development. To answer this question, adults completed an online survey about 11 central principles derived from three word learning theories (i.e., constraints, sociopragmatic, domain-general theories). Experiment 2 was designed to test whether the perceived difficulty of an item explained why adults had less accurate intuitions about principles derived from domain-general theories. Finally, Experiment 3 was designed to rule out that the test items for domain-general theories elicited different levels of confidence and interest than principles derived from other theories. Taken together, these experiments afforded an analysis of whether and why there are alignments between research findings and adults’ intuitions.

## Experiment 1

Experiment 1 examined whether adults’ intuitions about children’s word learning align with the scientific literature. For this purpose, a sample of undergraduate students, adults from the general public (non-parents), parents, and Speech Language Pathologists completed an online survey about central findings from early language development research. We predicted that adults would demonstrate less alignment on principles derived from domain-general theories. This prediction was drawn from prior studies demonstrating that adults have inaccurate perceptions of domain-general principles in memory (Kornell & Bjork, 2007, 2008). In addition, we predicted that parents and SLPs would have intuitions that were more aligned with research findings for all principles.

### Method

#### Participants

The participants included 89 undergraduate students, 93 adults from the general public, 88 parents, and 77 SLPs (see Table 2 for demographic information by participant group). A power analysis, based on the proportions reported in a conceptually similar study (Brewin et al., 2019), was conducted to determine a sample size that would provide at least 80% power. A power analysis for a chi-squared test of independence with α = 0.003 (corrected for multiple comparisons) yielded a sample size of 62 participants per group to achieve 80% power.

**Table 2:** Mean (SD) demographic information by participant group

Variable	Students (*n* = 89)	Public/non-parents (*n* = 93)	Public/parents (*n* = 88)	SLPs (*n* = 77)
Age (years)	19.92 (1.56)	34.81 (10.65)	41.69 (10.05)	35.44 (10.56)
Gender (male:female:other)	7:82:0	65:25:1	38:50:0	3:72:0
*Race*				
American Indian or Alaska Native	–	–	–	–
Asian	11.2%	8.6%	1.1%	6.5%
Black or African American	1.1%	6.5%	6.8%	3.9%
Native Hawaiian or Other Pacific Islander	1.1%	–	1.1%	–
White	80.9%	80.6%	90.9%	85.7%
More than one race	2.2%	3.2%	–	1.3%
Prefer not to disclose	3.4%	1.1%	–	2.6%
Ethnicity				
Hispanic or Latino	2.3%	5.4%	3.5%	9.2%
Not Hispanic or Latino	77.0%	92.5%	95.3%	78.9%
Prefer not to disclose	20.7%	2.2%	1.2%	11.8%
*Income*				
Less than $24,000	19.1%	15.1%	11.4%	9.1%
$25,000–$49,000	12.4%	39.8%	37.5%	9.1%
$50,000–$99,999	9%	35.5%	37.5%	39.0%
$100,000 or more	31.5%	7.5%	13.6%	32.5%
Prefer not to disclose	28.1%	2.2%	–	10.4%
Education Level				
Some high school	1.1%	–	–	–
High school graduate	2.3%	22.6%	14.8%	–
Some college	27.3%	19.4%	23.9%	–
Trade/technical/vocational	3.4%	10.8%	8.0%	–
College Graduate	29.5%	37.5%	42.0%	13.0%
Postgraduate	31.8%	9.7%	11.4%	87.0%
Prefer not to disclose	4.5%	–	–	–
Parenthood status (yes:no)	0:89	0:93	88:00	37:40
Average number of children	–	–	2.06 (0.98)	1.81 (1.17)
Average age of youngest child (years)	–	–	7.21 (7.31)	7.29 (8.75)

The survey was hosted using Qualtrics (www.qualtrics.com). The undergraduate participants signed up for the survey using a cloud-based participant management software (Sona Systems; https://www.sona-systems.com) and received course credit for their participation. The sample of adults from the general public (parents and non-parents) were recruited from Amazon’s Mechanical Turk (mTurk) and received $4.00 for completing the task. SLPs were recruited through the American Speech-Language-Hearing Association (ASHA) forum board and social media groups, and received a $10.00 gift card for their participation. An additional 27 participants were excluded from analyses because they failed one or both sound checks at the beginning of the survey.

#### Materials

All participants were administered a survey with 11 questions about word learning principles (Additional file 1: Supplementary Materials A). The word learning principles were derived from three major word learning theories, including constraints, sociopragmatic, and domain-general theories. These principles were selected because they are robust and replicable, suggesting that these word learning heuristics and mechanisms operate in children’s language development. The principles derived from constraints theories included the mutual exclusivity assumption (Markman & Wachtel, 1988), whole object assumption (Markman, 1991), taxonomic bias (Markman, 1991), and shape bias (Landau et al., 1988). The principles derived from sociopragmatic theories included learning from pointing (Booth et al., 2008), learning from looking to a visible object (Booth et al., 2008), learning from looking to a non-visible (or occluded) object (Baldwin, 1995), and overheard speech (Akhtar et al., 2001; Akhtar, 2005). Finally, principles derived from domain-general theories included learning from massed versus spaced presentation schedules (Vlach & Sandhofer, 2012a), cross-situational word learning (Smith & Yu, 2008), and learning in varied versus repeated contexts (Smith et al., 1978).

The word learning principles were presented the way children would encounter them if they came to the laboratory (and in the primary way these principles are tested in the literature). Although the tasks are largely laboratory-based, many of these principles were derived from observations of how children learn words in naturalistic environments and/or have generalized to naturalistic word learning environments. For instance, parents use pointing and eye-gaze in real-world settings, which has been linked to better language outcomes in children (e.g., Cartmill et al., 2010). Spaced schedules and variable contexts benefit learning in classrooms and clinical settings (e.g., Leonard et al., 2021, 2022; Sobel et al., 2011). Finally, cross-cultural studies have shown that children acquire language from overheard speech in their environment (e.g., Shneidman & Goldin-Meadow, 2012).

**Fig. 1:**
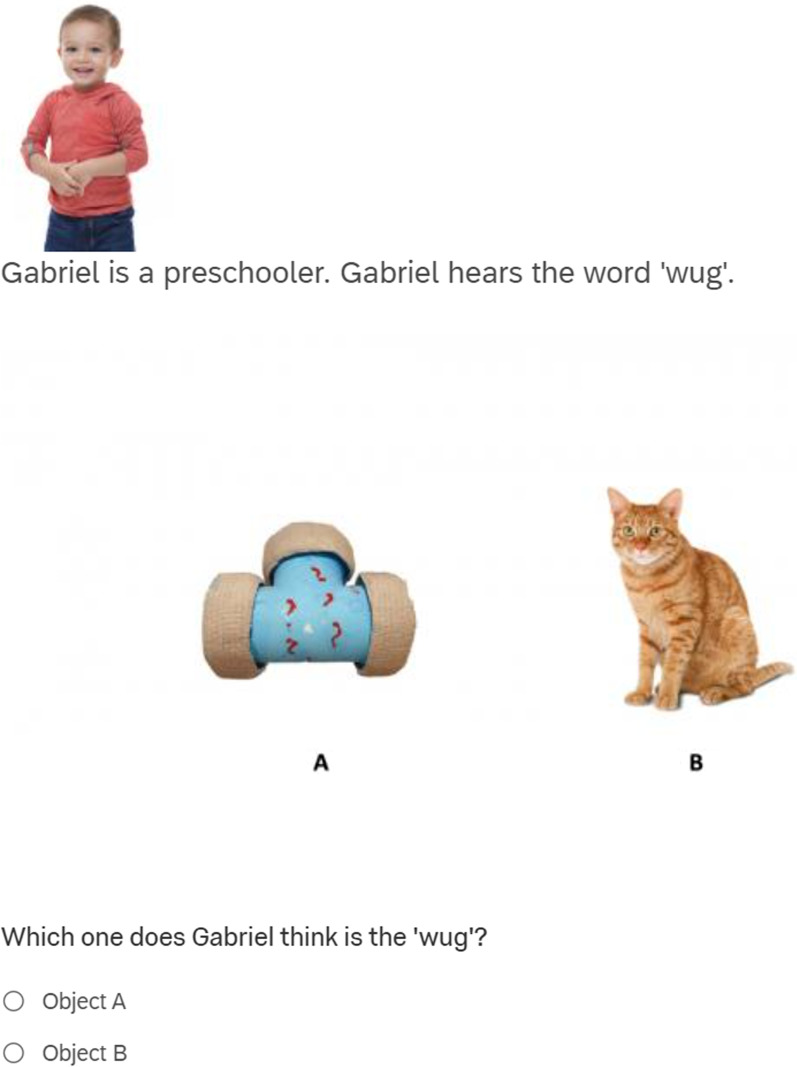
Example survey question for the “Mutual Exclusivity Assumption”

For each word learning principle, participants were introduced to a preschooler named Gabriel and were prompted to select an answer based on Gabriel’s learning environment. For example, when testing the mutual exclusivity assumption, participants were shown a familiar and a novel object and told: *“Gabriel is a preschooler. Gabriel hears the word “wug”. Which one does Gabriel think is the “wug”?”* (Fig. 1). We used this format as opposed to a generic question (e.g., *“A preschooler hears the word “wug”. Which one does the preschooler think is the “wug”?*) to facilitate taking the perspective of a preschooler. Participants also saw a picture of a preschooler who was around 3 years old. For principles derived from constraints theories, participants saw static images when answering the questions. For the principles derived from sociopragmatic and domain-general theories, participants viewed videos. The reason the formats differed is that word learning principles derived from sociopragmatic and domain-general theories either unfold across time or include an interactional component. Presenting videos therefore most closely resembled the way participants would encounter these tasks in the laboratory and in naturalistic contexts.

Finally, participants completed a questionnaire about relevant demographic information and language history. This survey also included questions about whether participants had children and the age of their children. For the SLP sample, we asked additional questions related to their training and clinical experience (Additional file 1: Supplementary Materials B and C, Table 1).

#### Procedure

All surveys were completed online after participants provided consent. The first two survey questions tested whether participants had functioning loudspeakers on their computers because six survey questions required sound (i.e., *“This study requires a functioning loudspeaker. Please turn on your sound and indicate what you hear in this sound clip.”*). The 11 word learning principles were shown after the sound check. Finally, participants completed the demographic and language survey.

#### Data Analysis

We used R (Version 3.5.1; R Core Team, 2021) and the *citr*, *ez*, *haven*, and *tidyverse* packages for all data processing and analyses. We started by calculating the percentage of participants whose intuitions aligned versus did not align with empirical results on the word learning principles. Then, we assessed group differences between adults with more experience with children’s language development (i.e., parents and SLPs) and adults with less experience (i.e., undergraduate students, general public/non-parents) using chi-squared tests of independence. In addition, we reported the results of a series of logistic regressions assessing the role of formal and informal experience with language development on alignment in the Additional file 1: Supplementary Materials D.

### Results and discussion

#### Participants’ intuitions

We began our analyses by calculating the percentage of participants whose intuitions aligned and did not align for each word learning principle. Results supported our hypotheses: Adults’ demonstrated a high degree of alignment with scientific findings for all principles except those derived from domain-general theories. For instance, collapsing across all groups, 93.37% of participants believed children would display mutual exclusivity, 95% CI [0.90, 0.96]. Similarly, most participants claimed that children display the whole object assumption (95.98%), 95% CI [0.93, 0.98], taxonomic bias (96.84%), 95% CI [0.94, 0.98], and shape bias (68.30%), 95% CI [0.63, 0.73]. For sociopragmatic theories of word learning, participants agreed that children’s language learning is supported with looking at (63.51%), 95% CI [0.58, 0.63], and pointing to (97.99%), 95% CI [0.96, 0.99], a visible object. Furthermore, most participants claimed that children could learn from overheard speech (80.69%), 95% CI [0.76, 0.84], and use eye-gaze to learn a label when the object was not visible (90.52%), 95% CI [0.87, 0.93].

In contrast, participants’ responses were more mixed for domain-general theories: A smaller percentage claimed that children could learn novel words cross-situationally (26.15%), 95% CI [0.22, 0.31], in varied contexts (35.92%), 95% CI [0.31, 0.41], and using a spaced presentation schedule (50.00%), 95% CI [0.45, 0.55].

#### The role of informal and formal experience

**Fig. 2:**
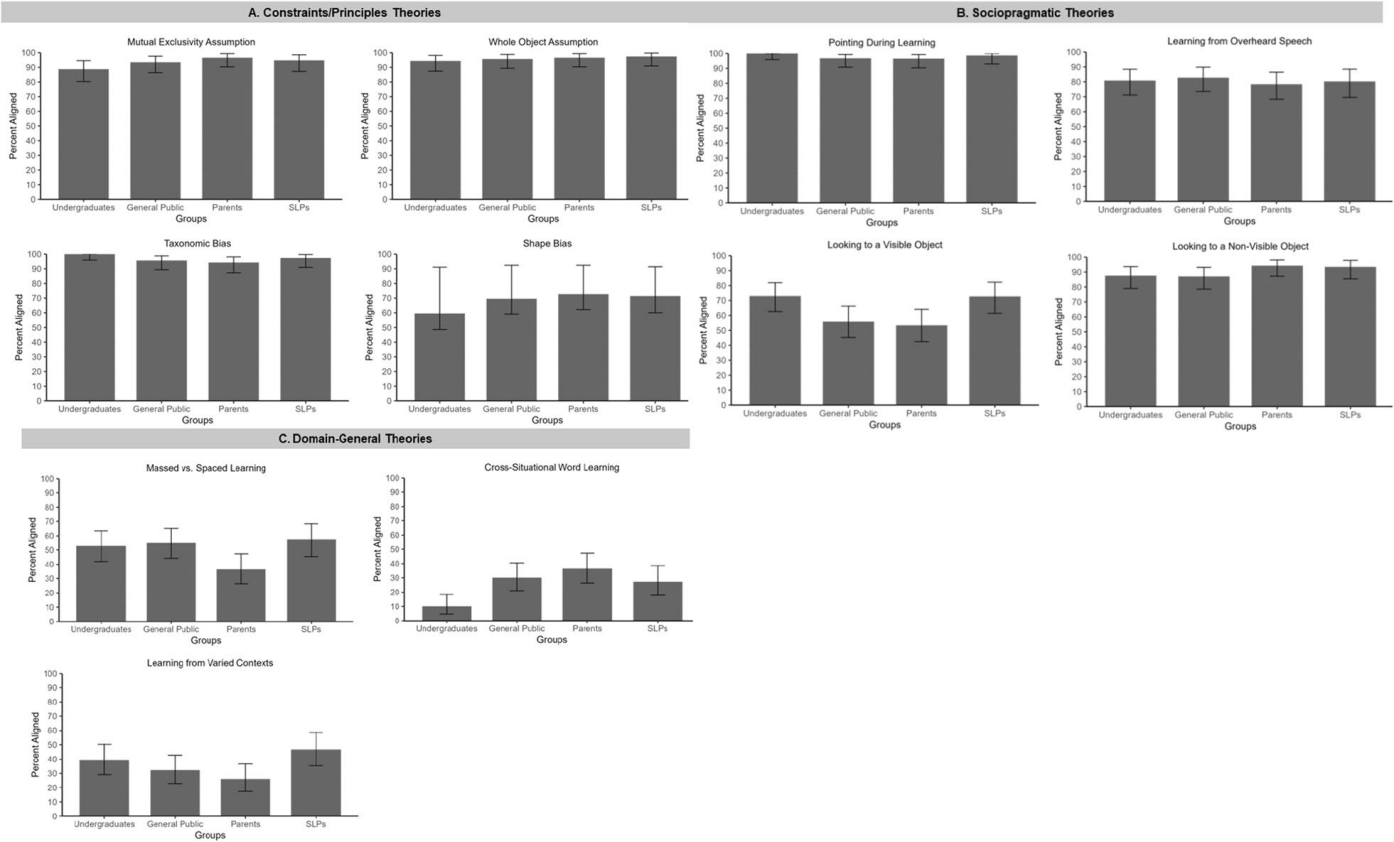
Percentage of participants whose intuitions aligned with research findings for each word learning principle by constraints (**A**), sociopragmatic (**B**), and domain-general (**C**) theories. Error bars represent 95% confidence intervals

Next, we were interested in whether the four participant groups (undergraduates, general public, parents, SLPs) differed in their intuitions regarding the word learning principles (Fig. 2); that is, whether informal or formal experience with language development shaped adults’ intuitions. We hypothesized that adults’ experience with children’s language learning or instruction might result in intuitions that are more closely aligned with empirical findings. To determine whether there were overall group differences in alignment with research findings, we first conducted a series of Bonferroni-corrected chi-squared tests of independence (15 comparisons; *α* = 0.05/15 = 0.003). Results revealed significant differences between groups for only the cross-situational word learning principle, ${\chi }^{2}=$ 17.50, *p* < 0.001. To isolate the nature of this difference, we conducted another series of chi-squared tests of independence comparing parents to undergraduates and the general public, and SLPs to undergraduates and the general public. We hypothesized that there may be differences between parents, who have informal experience with children’s word learning, as well as SLPs, who have formal experience with children’s word learning, and the other two participant groups. These analyses only revealed that parents (36.36%) had intuitions that were more aligned with research findings for cross-situational word learning than the undergraduates (10.11%), ${\chi }^{2}=$ 15.69, *p* < 0.003. All other comparisons were not significant.

Although parents accurately intuited that a preschooler could learn new words in a cross-situational word learning paradigm in comparison to undergraduate students, they still demonstrated a high degree of misalignment with expert consensus for all domain-general theory principles. These results suggest that informal or formal expertise with language development does not lead to intuitions that are more aligned with the scientific literature for these principles.

#### Amount of experience with children’s language development

Finally, we conducted analyses to determine whether the amount of parents’ and SLPs’ experience with children’s language development predicted alignment with research findings. We conducted a series of logistic regressions to assess the effect of several demographic factors within each group (e.g., age of youngest child, educational background, occupation, clinical expertise) on adults’ intuitions (see Additional file 1: Supplementary Materials D). These analyses revealed no significant impact of various demographic factors on adults’ intuitions, with one notable exception: SLPs who worked with young children were less likely to view spaced learning as better than massed learning, relative to SLPs who did not work with young children. This suggests that more experience with young children can lead to intuitions that are less aligned with research for the massed versus spaced learning principle.

#### Interim discussion

The results of Experiment 1 revealed that adults’ intuitions were aligned with research findings on principles derived from constraints and sociopragmatic theories. However, we observed a different pattern of results for domain general theories. The majority of participants did not demonstrate alignment with scientific consensus for principles. This was true across all groups regardless of adults’ experience with children or language learning, which leads us to our next research question: Why do adults have different intuitions about principles derived from domain-general theories?

Our first step in explaining the results from Experiment 1 was to consider differences between principles derived from domain-general theories and principles derived from the other theories. One key difference is that the learning conditions for principles derived from domain-general theories appear more difficult. Children must map words to objects and retain these mappings across several trials. Indeed, two domain-general theory principles from the present study—spaced learning and contextual variation—are drawn from *desirable difficulties* research. This body of work suggests that long-term retention of information is enhanced when learning conditions are optimally difficult (Bjork, 1994; Bjork et al., 2013; Kornell & Bjork, 2008).

Even though task difficulty may benefit long-term learning, adults view difficult learning conditions as undesirable and tend to avoid them (Miele et al., 2011; Simon & Bjork, 2001; Sungkhasettee et al., 2011). For instance, in spaced learning tasks, each presentation of an item is separated by a time lag. This time lag induces forgetting of the presented information and increases the effort necessary to retrieve forgotten items (Vlach, 2014, 2019). In contrast, no time lag is present in massed learning schedules, resulting in a *massed bias*: adults view massed schedules as more beneficial for learning than spaced schedules (e.g., Baddeley & Longman, 1978; Kornell & Bjork, 2007, 2008; McCabe, 2011; Vlach et al., 2019; Zechmeister & Shaughnessy, 1980).

Research on the massed bias demonstrates that adults have a subjective sense of task difficulty and view difficult learning conditions as disadvantageous for learning. It is plausible that adults also apply their metacognition of domain-general theory principles to preschoolers’ learning. That is, they may perceive principles derived from domain-general theories as more difficult and thus disadvantageous for children’s language learning. The goal of Experiment 2 was therefore to determine whether perceived task difficulty could explain adults’ intuitions on domain-general word learning principles.

## Experiment 2

Experiment 2 aimed to identify a mechanism underlying performance in Experiment 1. Specifically, this study assessed whether perceived task difficulty could explain adults’ intuitions for principles derived from domain-general theories. Participants completed the same online survey about central findings from research on early language development from Experiment 1. Following each word learning principle, participants were asked to rate how difficult the task would be for a preschooler. We predicted that participants would rate task difficulty higher for domain-general principles than other word learning principles.

### Method

#### Participants

A sample of 91 participants was recruited (90.11% female, 7.69% male) with a mean age of 19.92 years (*SD* = 1.29) (range: 18.00 to 25.00 years). We recruited undergraduate students because Experiment 1 showed no qualitative differences between undergraduate students, adults from the general public, parents, and SLPs. Participants reported being White (92.31%), Asian (2.20%), Black (2.20%), or more than one race (1.10%). The survey was hosted using Qualtrics (www.qualtrics.com). Participants signed up for the survey using a cloud-based participant management software (Sona Systems; https://www.sona-systems.com) and received course credit for their participation.

#### Material

The materials resembled Experiment 1, except for one difference: Participants were asked to rate the difficulty of the task for a preschooler after each of the 11 word learning principle questions. For example, after testing the mutual exclusivity assumption, participants were asked to *“Please rate how difficult it would be for Gabriel to do this task”* on a 5-point Likert scale (1 = “Not difficult at all; 5 = “Extremely difficult"). For principles that asked participants which of two conditions (i.e., pointing vs. not pointing, looking vs. not looking, same vs. varied contexts, massed vs. spaced) would help a preschooler learn new words, participants provided separate difficulty ratings for each option.

#### Procedure

The procedure resembled Experiment 1, except for the addition of difficulty ratings after each word learning principle.

#### Data analysis

We again examined the percentage of participants whose intuitions aligned vs. did not align with research findings for each word learning principle to assess whether the results from Experiment 1 would replicate. Then, we compared mean difficulty ratings by word learning theory using Bonferroni-corrected paired samples t-tests. Furthermore, we used a series of Bonferroni-corrected independent samples t-tests to assess whether difficulty ratings differed between participants whose intuitions aligned vs. did not align with research findings for each word learning principle.

### Results and discussion

#### Participants’ intuitions

We began our analyses by calculating the percentage of participants that selected each answer on the word learning principle questions. Results replicated Experiment 1: Adults’ intuitions matched scientific findings for all principles except those derived from domain-general theories. Most participants claimed that children display the mutual exclusivity assumption (95.60%), 95% CI [0.89, 0.98], the whole object assumption (95.98%), 95% CI [0.90, 0.98], the taxonomic bias (97.80%), 95% CI [0.92, 0.99], and the shape bias (70.33%), 95% CI [0.60, 0.79]. Participants also agreed that children’s language learning is supported with looking (73.63%), 95% CI [0.64, 0.82], and pointing (98.90%), 95% CI [0.94, 1.00], to a visible object. Additionally, most participants claimed that children could learn from overheard speech (78.02%), 95% CI [0.68, 0.85], and utilize eye-gaze when the target object was not visible (89.01%), 95% CI [0.81, 0.94].

Finally, participants’ responses were more mixed for domain-general theories. A smaller percentage claimed that preschoolers could learn novel words in cross-situational word learning paradigms (21.98%), 95% CI [0.15, 0.32], in varied contexts (35.16%), 95% CI [0.26, 0.45], and using a spaced presentation schedule (51.65%), 95% CI [0.42, 0.62].

#### Difficulty ratings

We predicted that perceived difficulty of a task might explain participants’ intuitions about whether a preschooler could complete the task. To test this prediction, we conducted a series of Bonferroni-corrected (3 comparisons; $\alpha =$ 0.05/3 = 0.016) paired samples t-tests comparing mean difficulty ratings across word learning theories. The results supported our prediction: Principles derived from domain-general theories (*M* = 2.76, *SD* = 0.48) were rated as significantly more difficult than principles derived from sociopragmatic theories (*M* = 2.49, *SD* = 0.65), *t*(90) = 4.32, *p* < 0.001, *d* = 0.47, and constraints theories (*M* = 2.12, *SD* = 0.55), *t*(90) = 8.67, *p* < 0.001, *d* = 1.24. In addition, principles derived from sociopragmatic theories were rated as significantly more difficult than principles derived from constraints theories, *t*(90) = 4.93, *p* < 0.001, *d* = 0.61.

Next, we assessed whether difficulty ratings differed based on whether participants’ intuitions matched research findings. A series of Bonferroni-corrected (15 comparisons; $\alpha =$ 0.05/15 = 0.003) independent samples t-tests showed that principles derived from domain-general and sociopragmatic theories were rated as significantly more difficult if the adults’ intuitions did not align with research findings than if the adults’ intuitions did align with research findings (Table 3).

**Table 3:** Mean (SD) difficulty ratings for each word learning principle by participants with aligned versus different intuitions

Variable	Aligned/different intuition on word learning principle				
	Aligned	Different	*t*	*p*	*d*
Mutual exclusivity	2.53 (1.00)	3.00 (0.53)	0.93	0.34	0.59
Whole object assumption	2.08 (1.08)	3.00 (1.58)	1.64	0.10	0.68
Taxonomic bias	1.42 (0.68)	2.00 (0.00)	1.19	0.24	1.21
Shape bias	2.25 (1.02)	2.68 (0.92)	1.83	0.07	0.44
Pointing	1.33 (0.65)	2.00 (–)	1.01	0.31	–
Not pointing	3.33 (0.91)	2.00 (–)	− 1.46	0.15	–
Looking to visible object	1.57 (0.61)	2.96 (0.75)	9.02	< 0.001*	2.03
Not looking to visible object	2.90 (0.80)	2.30 (0.70)	− 3.15	< 0.003*	0.80
Overheard speech	2.22 (0.81)	4.05 (0.68)	9.14	< 0.001*	2.44
Looking to non-visible object	3.04 (1.13)	3.60 (0.84)	1.52	0.13	0.56
Massed learning	2.96 (0.78)	1.82 (0.50)	− 8.26	< 0.001*	1.74
Spaced learning	1.91 (0.46)	3.34 (0.68)	11.80	< 0.001*	2.46
CSWL	2.90 (0.97)	4.24 (0.62)	7.46	< 0.001*	1.65
Varied context	1.94 (0.67)	3.22 (0.83)	7.50	< 0.001*	1.70
Same context	2.72 (0.92)	1.69 (0.70)	− 5.93	< 0.001*	1.26

Difficulty ratings by aligned and different intuitions for each word learning principle using a 1–5 Likert scale (1 = “Not difficult at all”, 5 = “Extremely difficult”). A series of paired samples *t*-tests, Bonferroni-corrected for multiple comparisons (α = 0.003), assessed differences in mean difficulty ratings between participants with aligned vs. misaligned responses, *p < 0.003. Only one participant answered incorrectly on the principle testing the importance of pointing during learning. As a result, no standard deviation is reported for this principle

Thus, the results from Experiment 2 revealed that perceived task difficulty was related to adults’ reasoning about word learning processes. Domain-general theory principles were rated as more difficult overall, especially when adults’ intuitions did not align with research findings (e.g., participants thought a preschooler could not learn cross-situationally). Sociopragmatic theory principles were also rated as more difficult than constraints theory principles. One explanation for this finding is that the sociopragmatic theory principles require children to make sophisticated inferences about the referential intent of others. This is likely considered a difficult task for children (e.g., Verbrugge, 2009). It is important to note that although sociopragmatic tasks were rated as more difficult when adults’ intuitions did not align with research findings, the number of participants whose intuitions were misaligned was lower than for domain-general theory principles. Taken together, these findings suggest that adults use task difficulty as a guide of what may or may not aid children’s language learning.

Before concluding that perceived task difficulty explains adults’ intuitions on the word learning principles, we asked whether there is an alternative mechanism to explain the results from Experiments 1 and 2. One possibility is that the domain-general theories evoked different affective experiences for participants. For instance, participants might have been confident in their responses on domain-general theory questions, as perceived task difficulty can influence confidence (e.g., Chung & Monroe, 2000; Kebell et al. 1996). Moreover, participants might also have viewed the domain-general theory questions as more or less interesting than questions derived from sociopragmatic or constraints theories, as perceived task difficulty may change level of interest (Fulmer & Tulis, 2013; Fulmer et al., 2015). If adults were using the level of confidence evoked by the tested principles to guide their responses, differences in confidence levels should emerge between adults whose intuitions aligned versus did not align for principles derived from domain-general theories. Similarly, if adults were using their interest level in a principle to guide their responses, differences in interest levels should emerge.

We sought to test these possibilities in Experiment 3. Participants provided their perceived confidence in their answer and their level of interest in learning more about each tested word learning principle. We predicted that confidence and interest levels would not differ between adults whose intuitions aligned versus did not align with research findings for principles derived from domain-general theories, thereby providing evidence against affective factors as an alternative mechanism to explain adults’ intuitions.

## Experiment 3

Experiment 3 was designed to replicate Experiments 1 and 2 and to assess whether confidence or interest may be contributing to adults’ intuitions. For this purpose, adults were presented with the same word learning principles from Experiments 1 and 2. For each word learning principle, adults rated their perceived confidence in their answer and their interest in learning more about the principle. If confidence and interest are not driving intuitions on the word learning principles, we predicted that confidence and interest levels would not differ between theories and between adults whose intuitions aligned versus did not align for principles derived from domain-general theories.

### Method

Each participant completed the same online survey about word learning principles. Following each word learning principle, participants were asked to rate their confidence in their answer and their interest in learning more about the presented task. Finally, information about relevant demographics was collected.

#### Participants

A sample of 97 undergraduate students was recruited (83.51% female, 15.46% male, 1.03% non-binary) with a mean age of 19.51 years (*SD* = 1.32) (range: 18.00 to 26.00 years). Participants reported being White (86.60%), Asian (4.12%), Black (2.10%), or more than one race (4.12%). The survey was hosted using Qualtrics (www.qualtrics.com). Participants were recruited and compensated in the same manner as Experiments 1 and 2.

#### Materials

The materials resembled Experiment 1, except for one difference: After each of the 11 word learning principle questions, participants were asked to rate how confident they were in their answer on a 5-point Likert scale (1 = “Not confident at all”; 5 = “Extremely confident”). In addition, participants were asked to *“Please rate how interested [they] would be in learning more about this topic”* on a 5-point Likert scale (1 = “Not interested at all; 5 = ”Extremely interested”).

#### Procedure

The procedure resembled Experiments 1 and 2, except for the additional confidence and interest ratings following each word learning principle.

#### Data analysis

Much like Experiments 1 and 2, we first calculated the percentage of participants whose answers aligned versus did not align with research findings for each word learning principle. Then, we assessed differences in mean confidence and interest ratings between theories and between participants whose intuitions aligned versus did not align with research findings using t-tests.

### Results and discussion

#### Participants’ intuitions

To determine whether the findings from Experiments 1 and 2 replicated in the present sample, we tested the total percentage of individuals whose intuitions matched scientific findings. The results replicated our previous results: Adults’ intuitions matched scientific findings for all principles except those derived from domain-general theories. Much like Experiments 1 and 2, 90.72% of participants claimed that children display the mutual exclusivity assumption, 95% CI [0.83, 1.00]. Most participants claimed that children display the whole object assumption (89.69%), 95% CI [0.82, 0.94], taxonomic bias (100.00%), 95% CI [0.96, 1.00], and shape bias (62.89%), 95% CI [0.53, 0.72]. Participants also agreed that children’s language learning is supported with looking (72.16%), 95% CI [0.63, 0.80], and pointing (98.97%), 95% CI [0.94, 1.00], to a visible object. Furthermore, most participants claimed that children could learn from overheard speech (83.51%), 95% CI [0.75, 0.89], and utilize eye-gaze when the target object was occluded (89.69%), 95% CI [0.82, 0.94].

Participants’ responses were again more mixed for domain-general theories. A smaller percentage claimed that preschoolers could learn novel words in cross-situational word learning paradigms (28.87%), 95% CI [0.21, 0.39], in varied contexts (43.30%), 95% CI [0.34, 0.53], and using a spaced presentation schedule (52.58%), 95% CI [0.43, 0.62].

#### Confidence and interest ratings

We also predicted that confidence and interest levels would not significantly differ between the three theories. To test this prediction, we calculated the mean confidence and interest ratings for each word learning theory. We then conducted a series of Bonferroni-corrected paired samples t-tests (4 comparisons; $\alpha =$ 0.05/4 = 0.0125) comparing mean confidence and interest ratings for domain-general theories with sociopragmatic and constraints theories. Results revealed that mean confidence ratings for principles derived from domain-general theories (*M* = 2.95, *SD* = 0.71) did not differ significantly from principles derived from constraints theories, (*M* = 2.89, *SD* = 0.73), *t*(96) = 0.75, *p* = 0.45, *d* = 0.08. However, participants did report significantly lower confidence ratings for principles derived from domain-general theories than sociopragmatic theories (*M* = 3.15, *SD* = 0.72), *t*(96) = − 2.91, *p* = 0.004, *d* = 0.28. Mean interest ratings for principles derived from domain-general theories (*M* = 2.96, *SD* = 0.80) were significantly lower than constraints theories (*M* = 3.09, *SD* = 0.76), *t*(96) = − 2.76, *p* = 0.007,* d* = 0.17, yet did not differ significantly from sociopragmatic theory principles (*M* = 2.86, *SD* = 0.84), *t*(96) = 1.86, *p* = 0.066, *d* = 0.12.

Next, we assessed whether confidence and interest ratings differed based on whether participants’ intuitions matched research findings. For this purpose, we calculated the mean confidence and interest ratings for each word learning principle (Additional file 1: Supplementary Materials C, Table 2 and Table 3). It is important to note that certain principles (e.g., pointing during learning, taxonomic bias) did not have enough participants whose intuitions did not align with research findings to make meaningful comparisons. We conducted a series of Bonferroni-corrected independent samples t-tests (15 comparisons; $\alpha =$0.05/15 = 0.003) comparing mean confidence ratings between participants based on whether their intuitions aligned or did not align with research findings; results revealed no significant differences in mean confidence ratings, *ps* > 0.003. Similarly, a series of Bonferroni-corrected independent samples t-tests (15 comparisons; $\alpha =$ 0.05/15 = 0.003) revealed no significant differences in mean interest ratings between adults whose intuitions aligned or did not align with research findings, *ps* > 0.003.

Taken together, these results suggest that domain-general theories did evoke slightly different affective experiences. Specifically, participants were less confident in their responses for domain-general theory principles than sociopragmatic theory principles, and less interested in learning more about the domain-general theory principles than constraints theory principles. Although domain-general theory principles evoked slightly different affective responses, confidence and interest ratings did not differ between adults whose intuitions aligned versus did not align with research findings. This suggests that personal affective experiences during the survey were unlikely driving adults’ intuitions about domain-general theory principles.

## General discussion

The purpose of this study was to assess adults’ intuitions about research on children’s word mapping and learning. Experiment 1 revealed that adults’ intuitions aligned with research findings for all principles except for those derived from domain-general theories. Experiment 2 revealed that perceived difficulty of a task for a preschooler may be a mechanism underlying adults’ intuitions. Finally, Experiment 3 ruled out other item-related factors, such as perceived interest and confidence, as possible explanations. Taken together, this work suggests that although adults have an accurate sense of how children learn words, they view difficult learning conditions as undesirable for children’s word learning.

A key takeaway from Experiment 1 is that adults’ intuitions were largely aligned with research findings; that is, they had an accurate sense of how preschoolers—on average—respond to these laboratory tasks. Most adults predicted that children would attribute a novel label to the whole object, a shape match, and a taxonomic match (constraints theory principles). Most adults also agreed that using eye gaze and pointing would support children’s ability to map novel words to objects (sociopragmatic theory principles). This pattern held across the four samples recruited for Experiment 1. Adults can thus form aligned intuitions about children’s language learning through informal (e.g., having been a child, interactions within the family, babysitting) and formal (e.g., clinical training) experiences, and constraints and sociopragmatic theory principles do not need to be explicitly taught. Adults’ intuitions were less aligned with research findings for principles derived from domain-general theories. For instance, adults were less certain whether children could learn novel words cross-situationally, on a spaced schedule, or in varied contexts. These results also held across all four samples, suggesting that prior experience with children’s language learning or language instruction does not lead to more aligned intuitions for domain-general theory principles.

Speech Language Pathologists—who have the most formal experience with language development—did not demonstrate more alignment than the other participant groups. This is surprising considering that domain-general theories of language learning have been used to explain intellectual and developmental disabilities (see Saffran, 2018 for a review). For instance, developmental language disorder has been attributed to difficulties in domain-general mechanisms, such as implicit and statistical learning (Benham et al., 2018; Evans et al., 2009; McGregor et al., 2017; Ullman & Pierpont, 2005). As such, domain-general tasks, like the cross-situational word learning paradigm, have been used to study developmental language disorder, apraxia of speech, aphasia, and late talking (Ahufinger et al., 2021; McGregor et al., 2022; Peñaloza et al., 2017). Moreover, several domain-general theory principles (e.g., spaced and variable learning) have been effectively applied to intervention work in clinical research settings (Desmottes et al., 2017; Haebig et al., 2019; Leonard et al., 2021, 2022; Levlin et al., 2022; Plante et al., 2014). Why did SLPs nonetheless demonstrate similar alignment with the other participant groups? One explanation is that domain-general learning theories are valuable for understanding the etiology of language disorders, but are more difficult to implement in intervention work. Indeed, domain-general theory principles are not typically applied to interventions and little guidance for implementation currently exists (see Alt et al., 2012; Justice et al., 2016; Plante & Gómez, 2018, for suggestions; Steele, 2020). Possible barriers to implementation include disagreements about the underlying etiologies of disorders and questions about how to adapt these principles for children with intellectual and developmental disorders during treatment sessions (Alt et al., 2012; Baron & Arbel, 2022). Another significant barrier is that these principles are counterintuitive; that is, they contradict conventional clinical guidance, which is to move from simple to more complex structures (Alt et al., 2012). Thus, applying domain-general principles to intervention work will require communicating the value of difficult learning conditions for learning outcomes and developing clear guidance for clinicians.

Adults across all participant groups were less aligned for domain-general theory principles. One hypothesis is that adults with/without prior experience with children’s language development view these principles as more difficult than principles derived from constraints or sociopragmatic theories. Indeed, Experiment 2 supported this hypothesis: Adults rated domain-general tasks as more difficult for a preschooler to complete than constraints and sociopragmatic tasks. Moreover, perceived difficulty impacted whether adults thought a preschooler could complete the task. If adults thought a preschooler could not perform the task, they rated the task as more difficult than adults who thought a preschooler could perform the task.

These findings suggest that adults apply their bias against difficult learning conditions to children, even though children might benefit from these conditions (Kornell & Bjork, 2007, 2008; McCabe, 2011; Vlach et al., 2008; Vlach & Sandhofer, 2012a, b). If adults view difficult learning conditions, such as spaced learning, as undesirable for their own learning, it is not surprising that they would view difficult learning conditions as undesirable for children. The bias against difficult learning conditions may be even more pronounced for children because adults tend to underestimate children’s abilities (e.g., Miller et al., 1980). Furthermore, there are likely to be developmental and individual differences in children’s ability to learn from difficult learning conditions (see Knabe & Vlach, 2020, for a discussion of this issue). Future studies should therefore assess the conditions under which adults view difficult learning conditions as undesirable or desirable for children.

Another explanation for the bias is that adults are more accurate in estimating children’s abilities when they are directly observable (e.g., the words children say) than when they are not directly observable (e.g., memory span; Holden & Smith, 2019; Miller & Davis, 1992). Indeed, the paradigms used to study constraints and sociopragmatic theories were likely drawn from observable experiences with how children make inferences in-the-moment. In comparison, domain-general theory principles rely on learning mechanisms that are not directly observable, that are internal to the learner, and that unfold across time. For instance, in cross-situational word learning tasks, children must track the co-occurrence of words and objects across time (Smith & Yu, 2008, 2013; Vlach & Johnson, 2013). Although adults can know which objects children see and which words they hear, they do not necessarily know if children successfully made word mappings from this experience. Information about whether a child has successfully mapped words to their intended referents is located solely in the mind of the child. In brief, adults might make inaccurate predictions about children’s performance when learning outcomes are largely unobservable.

A final explanation for the bias against domain-general theory principles is that adults use their own word learning strategies to respond to the scenarios. That is, the word mapping and learning scenarios presented in Experiments 1–3 might not test adults’ intuitions of how a preschooler would behave in-the-moment. Instead, the responses to the scenarios might reveal adults’ intuitions about how they would behave in-the-moment. Prior work has found that adults use fundamentally different word learning strategies than children and assume that children adopt the same strategies (Ramscar et al., 2013). It is therefore possible that adults used their own word learning strategies to predict children’s behavior in the tasks, leading to misalignment with the domain-general theory principles. Future studies will therefore be necessary to identify the relative contribution of these factors to adults’ intuitions about domain-general theory principles.

The present study focused on adults’ intuitions about how children behave in word mapping and learning scenarios, revealing a high degree of misalignment with domain-general learning principles. Are adults who view these tasks as too difficult also more likely to avoid presenting children with difficult learning conditions? The link between beliefs and behaviors is a longstanding research area in social science (e.g., Bornstein et al., 2017). Prior research has shown that adults make assumptions about children’s cognition and adjust their behavior based on these assumptions (e.g., linguistic tuning hypothesis; Jensen de López et al., 2021; MacPhee, 1983; Sigel & McGillicuddy-De Lisi, 2002; Snow, 1972; Yurovsky et al., 2016). Their beliefs and behaviors surrounding children’s language development can even influence children’s long-term language outcomes (Bojczyk et al., 2016; Donahue et al., 1997; Del Vecchio et al., 2014; Hunt & Paraskevopoulos, 1980; Jimerson & Bond, 2001; Winstanley et al., 2014; Zippert & Ramani, 2017). Nonetheless, future research is needed to definitively answer whether adults’ intuitions about word learning shape children’s language development.

If we assume that there is a link between adults’ intuitions about word learning and their behavior, as well as a link between their behavior and children’s language outcomes, it is critical to instruct adults on how children learn words. The present study takes a first step in this direction by revealing an important area of knowledge misalignment for adults: the value of incorporating difficult learning conditions, such as domain-general mechanisms, in language instruction. Domain-general theory principles have been shown to apply to naturalistic word learning (e.g., Goldenberg et al., 2022; Slone et al., 2023), suggesting that adults’ metacognition of the tested word learning principles may indeed matter for early word learning. Addressing this misalignment is not an easy task as adults continue to hold biases against difficult learning conditions, even after explicit instruction about their benefit for learning (e.g., Kornell & Bjork, 2007; Kornell et al., 2010; Sungkhasette et al., 2011). Developing effective materials for public outreach or clinical training therefore presents a challenge for researchers. Prior to developing these materials, researchers should study how to address adults’ assumptions about how people learn (Kowalski & Taylor, 2009; McCabe, 2011; Teichert & Stacy, 2002). Once researchers understand how to successfully address these assumptions, intervention materials for parents, clinicians, and the general public can be developed.

In sum, the present study is the first to report incongruencies between adults’ intuitions and research findings on children’s word mapping and learning. Specifically, results revealed that adults mostly hold intuitions that align with research findings but are consistently misaligned on domain-general principles. One explanation for these intuitions is that domain-general principles are perceived as more difficult, and adults do not think difficult learning conditions are beneficial for learning. This intuition contradicts a rich literature on the efficacy of difficult learning conditions for learners (e.g., Kornell & Bjork, 2008; Roediger & Karpicke, 2006; Vlach et al., 2008). Considering these discrepancies, we urge researchers to assess what the public knows and does not know about children’s thinking and learning. Comparing adults’ intuitions to research findings that scientists consider robust is a critical first step in understanding how adults’ beliefs impact their behavior, and how their behavior impacts children’s outcomes. Moreover, understanding adults’ cognition about children’s learning will help scientists develop an evidence-based plan for disseminating research. These efforts will lay the groundwork for effective public engagement and ultimately benefit the key stakeholders of children’s language development.

## Supplementary Information

**Additional file 1.** **Supplementary Materials A:** Word Learning Principles by Theory. **Supplementary Materials B:** Questionnaire for All Samples. **Supplementary Materials C:** Table 1: Educational and Clinical Experience of SLP Sample. **Supplementary Materials D:** Exploratory Analyses.

## Data Availability

The datasets, analysis code, and research materials supporting the conclusions of this article are available at https://osf.io/3v6x4/?view_only=5843263610f64dceb7e1ef235c6b050a. This study’s design and its analysis were not pre-registered.
