# Supplementary material for: Does the public know what researchers know? Perceived task difficulty impacts adults’ intuitions about children’s early word learning
Source: Cogn Res Princ Implic. 2023 Jul 24;8:45. doi: 10.1186/s41235-023-00493-y (PMC10366060; doi:10.1186/s41235-023-00493-y)
Supplement: Supplementary file 1 — Additional file 1. Supplementary Materials A: Word Learning Principles by Theory. Supplementary Materials B: Questionnaire for All Samples. Supplementary Materials C: Table 1: Educational and Clinical Experience of SLP Sample. Supplementary Materials D: Exploratory Analyses. [file 41235_2023_493_MOESM1_ESM.docx]

**Supplementary Materials A**

Word Learning Principles by Theory

**
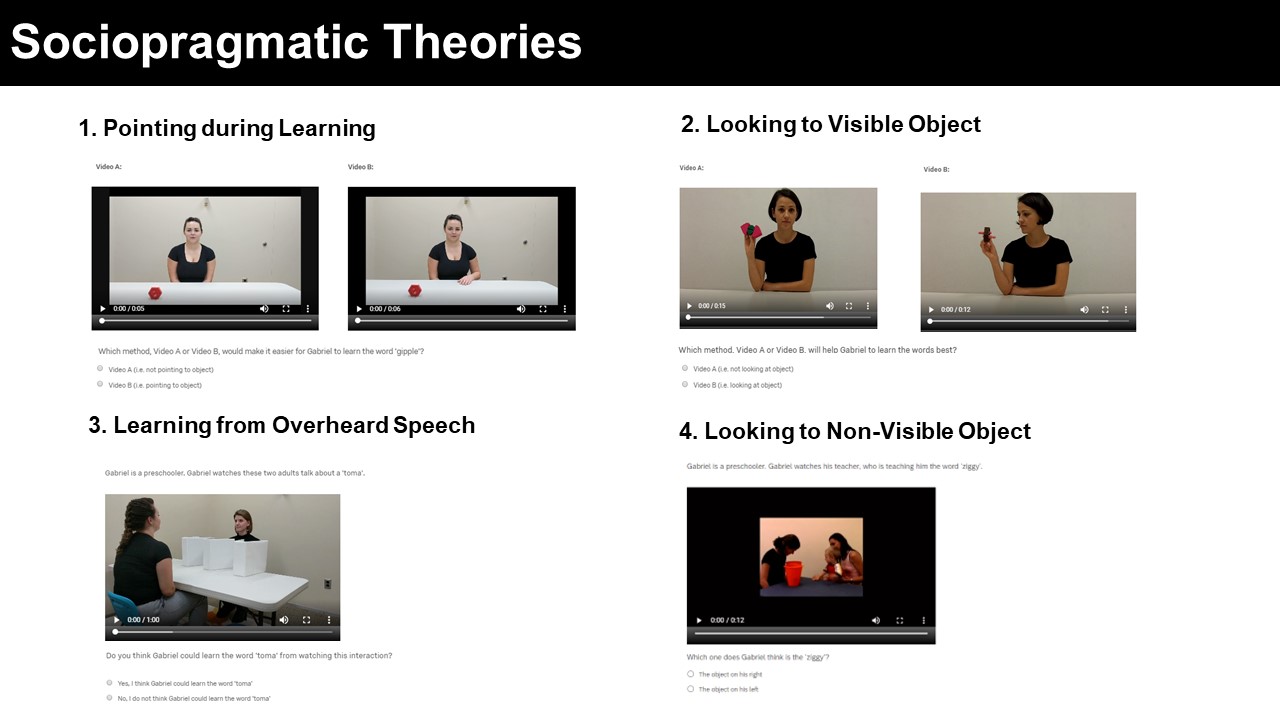

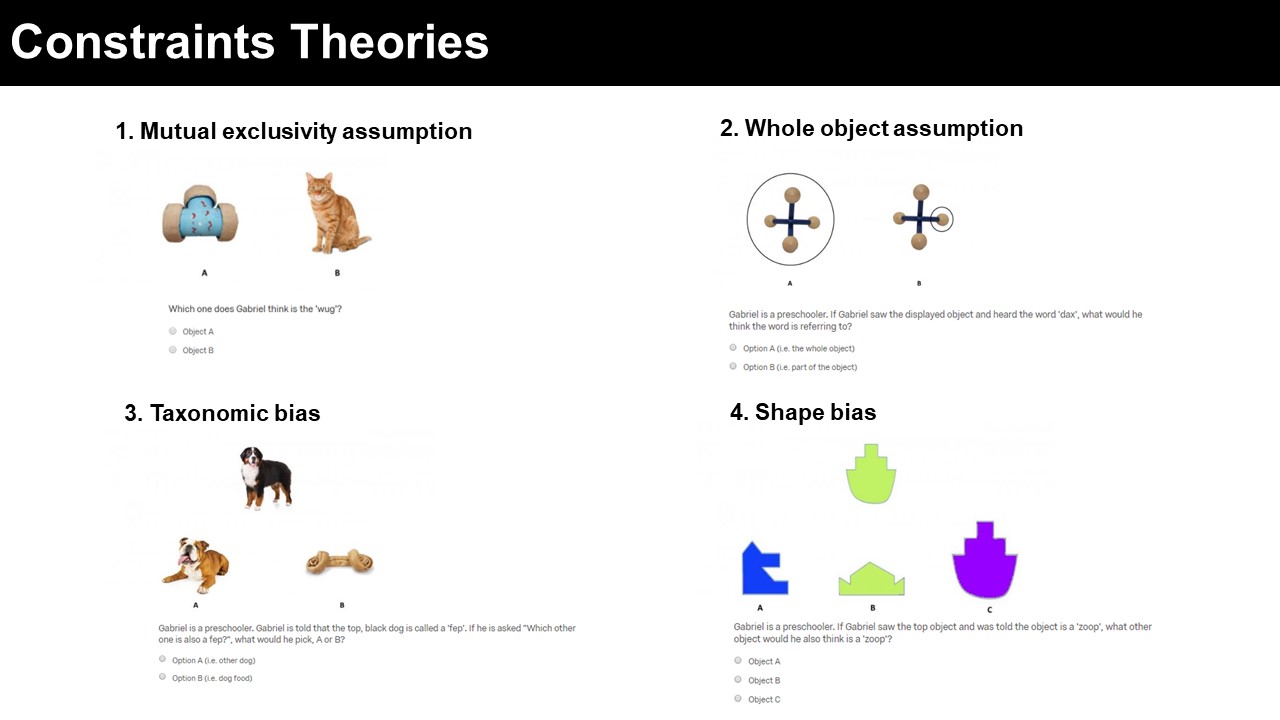

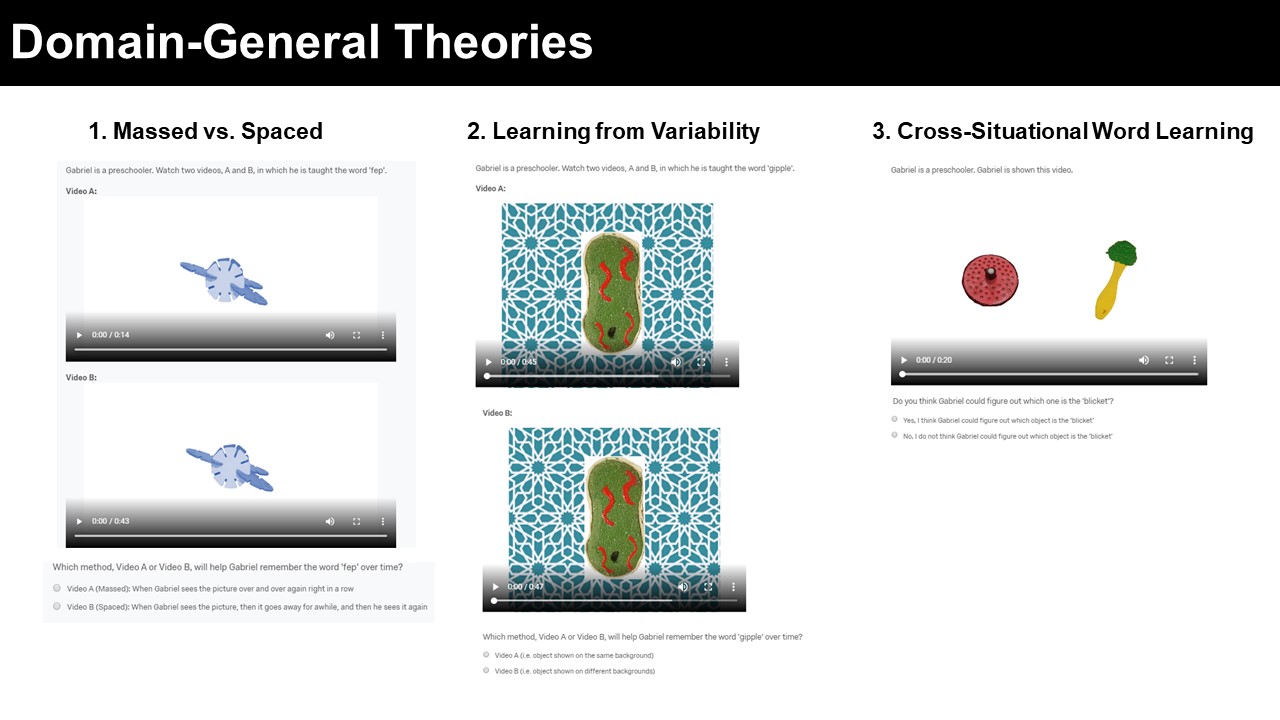
**

**Supplementary Materials B**

Questionnaire for All Samples

1. Gender. What is your gender
   1. Male
   2. Female
   3. Gender non-conforming/nonbinary
   4. Prefer not to disclose
2. Race. With which race do you most closely identify? Please select one:
   1. American Indian or Alaska Native
   2. Asian
   3. Black or African American
   4. Native Hawaiian or Other Pacific Islander
   5. White
   6. More than one race
   7. Prefer not to disclose
3. Ethnicity. With which ethnicity do you most closely identify? Please select one:
   1. Hispanic or Latino
   2. Not Hispanic or Latino
   3. Prefer not to disclose
4. Household income. What is your total household income? Please select one:
   1. Less than $24,999
   2. $25,000 to $49,999
   3. $50,000 to $99,999
   4. $100,000 or more
   5. Prefer not to disclose
5. Parental Education. What is the highest level education achieved in your household? Please select one:
   1. Some high school
   2. High school graduate
   3. Some college
   4. Trade/Technical/Vocational training
   5. College graduate
   6. Postgraduate
   7. Prefer not to disclose
6. Industry. Which of the following categories best describes the industry you primarily work in (regardless of your actual position)?
   1. Retired
   2. Unemployed
   3. Agriculture, Forestry, Fishing and Hunting
   4. Utilities
   5. Computer and Electronics Manufacturing
   6. Wholesale
   7. Transportation and Warehousing
   8. Software
   9. Broadcasting
   10. Other Information Industry
   11. Real Estate, Rental and Leasing
   12. Primary/Secondary (K-12) Education
   13. Health Care and Social Assistance
   14. Hotel and Food Services
   15. Homemaker
   16. Religious
   17. Mining
   18. Construction
   19. Other Manufacturing
   20. Retail
   21. Publishing
   22. Telecommunications
   23. Information Services and Date Processing
   24. Finance and Insurance
   25. College, University, and Adult Education
   26. Other Education Industry
   27. Arts, Entertainment, and Recreation
   28. Government and Public Administration
   29. Scientific or Technical Services
   30. Military
   31. Other Industry
7. What is your age?
8. Which of the following best describes the area you live in?
   1. Rural
   2. Urban
   3. Suburban
9. Which regions of the country do you live in?
   1. Midwest (IA, IL, IN, KS, MI, MO, ND, NE, OH, SD, WI)
   2. Northeast (CT, DC, DE, MA, MD, ME, NH, NJ, NY, PA, RI, VT)
   3. Southeast (AL, AR, FL, GA, KY, LA, MS, NC, SC, TN, VA, WV)
   4. Southwest (AZ NM, OK, TX)
   5. West (AK, CA, CO, HI, ID, MT, NV, OR, UT, WA, WY)
10. Multilingualism. Do you speak more than one language?
    1. Yes
    2. No
11. Language History. Which language(s) do you speak?
    1. English
    2. Spanish
    3. Mandarin
    4. Other: ___________________________
12. Please list the languages that you know. Rate your ability to listen to (i.e., understand) and speak the language on a scale from 1-7: (1) very poor; (2) poor; (3) fair; (4) functional; (5) good; (6) very good; (7) native-like

Native language: ___________ Listening Ability:_______ Speaking Fluency:______

Second language:___________ Listening Ability:_______ Speaking Fluency:______

Third language:____________ Listening Ability:_______ Speaking Fluency:______

1. Please specify at what age (in years) you started to learn your second language?
2. How did your child learn their second language?
   1. Mainly through formal classroom instruction
   2. Mainly through interacting with people
   3. A mixture of both (a) and (b)
   4. Other (specify):
3. What language(s) are spoken at home?_____________________
4. What language(s) are spoken at daycare/preschool/etc.?____________________
5. Estimate, in terms of percentages, how often you use your native language and other languages per day (in all daily activities combined). Total should equal 100%:
   1. Native language _____%
   2. Second language_____%
   3. Other languages______%
6. Has you lived in a country other than the United States?
   1. Yes (specify):__________________________
   2. No
7. Which concepts from this survey were familiar to you?
8. Do you study early word learning?
   1. Yes
   2. No
9. Do you have children?
   1. Yes
   2. No
10. How many children do you have (enter number below): ________
11. How old is/are your child/ren?
    1. Child 1: _______
    2. Child 2: _______
    3. Child 3: _______
    4. Child 4: _______
    5. Child 5: _______

Questionnaire for Speech-Language Pathologists

1. Are you currently a graduate student?
   1. Yes
   2. No

*Logic branch will show different questions, depending on the answer to Q1.*

**NOT A GRADUATE STUDENT**

1. What is the highest degree you have earned?
   1. Bachelor’s degree
   2. Master’s degree
   3. Doctoral degree (e.g., PhD)
      1. In a CSD discipline
      2. If outside a CSD discipline, please specify the discipline: _____
   4. Clinical doctorate
2. What year did you receive your highest degree? _____
3. Where did you receive your highest degree?
   1. Fill in blank for name of university
   2. Fill in blank for country
4. Are you certified in your field?
   1. Yes
   2. No
5. What is your primary employment facility (select all that apply)?
   1. Educational facility
      1. Early intervention
      2. Preschool
      3. K-12
      4. College/University
   2. Health care facility
      1. Hospital
      2. Residential health care facility
      3. Nonresidential health care facility
   3. Private practice
   4. Local, state, and federal governmental agencies
   5. Research/scientific organization
   6. Not employed
   7. Other: _______
6. Please select all work roles that apply to you:
   1. Clinical service provider
   2. Clinical supervisor
   3. Researcher
   4. Educator
   5. Postdoctoral fellow
   6. Graduate student
   7. Research assistant
   8. Consultant
   9. Retired
   10. Unemployed
   11. Other
7. Please specify the age groups you primarily work with (select all that apply):
   1. Birth to 3 years
   2. Preschool
   3. Elementary School-aged
   4. Middle School-aged
   5. High School-aged
   6. College/University-aged
   7. Adults
   8. Seniors
   9. Other
8. Please select all the clinical disorders you work with:
   1. Aphasia
   2. Apraxia of speech
   3. Autism
   4. Developmental language disorders
   5. Cleft lip and cleft palate
   6. Cognitive-communication disorders
   7. Dementia
   8. Dysarthria
   9. Dysphagia (adult)
   10. Dysphagia (pediatric)
   11. Fluency disorders
   12. Intellectual disabilities (e.g. Down Syndrome, Fragile X)
   13. Preschool language disorders
   14. Social communication disorder
   15. Selective mutism
   16. Speech sound disorders: articulation and phonology
   17. Voice and voice disorders

**FACULTY MEMBERS**

1. If you are employed at an institution of higher education, what is your academic rank?
   1. Full professor
   2. Associate professor
   3. Assistant professor
   4. Adjunct
   5. Instructor/Lecturer
   6. Other (e.g., research scientist)
   7. Do not hold academic appointment

**GRADUATE STUDENT**

1. What is the highest degree you have earned?
   1. Bachelor’s degree
   2. Master’s degree
   3. Doctoral degree (e.g., PhD)
      1. In a CSD discipline
      2. If outside a CSD discipline, please specify the discipline: _____
   4. Clinical doctorate
2. What is the highest degree you plan to earn?
   1. Bachelor’s degree
   2. Master’s degree
   3. Doctoral degree (e.g., PhD)
      1. In a CSD discipline
      2. If outside a CSD discipline, please specify the discipline: _____
   4. Clinical doctorate
3. What year are you projected to receive your highest degree? _____
4. Where are you working towards your highest degree?
   1. Fill in blank for name of university
   2. Fill in blank for country
5. Are you certified in your field?
   1. Yes
   2. No
6. Where has your clinical training primarily occurred?
   1. In-house
   2. Out-of-house
   3. Both
   4. NA
   5. Other: ____
7. Please select all the locations where you have received clinical training during your program:
   1. Educational facility
      1. Early intervention
      2. Preschool
      3. K-12
      4. College/University
   2. Health care facility
      1. Hospital
      2. Residential health care facility
      3. Nonresidential health care facility
   3. Private practice
   4. Local, state, and federal governmental agencies
   5. Research/scientific organization
   6. Not employed
   7. Other: _______
8. Please specify your desired primary employment facility after receiving your highest degree (select all that apply)?
   1. Educational facility
      1. Early intervention
      2. Preschool
      3. K-12
      4. College/University
   2. Health care facility
      1. Hospital
      2. Residential health care facility
      3. Nonresidential health care facility
   3. Private practice
   4. Local, state, and federal governmental agencies
   5. Research/scientific organization
   6. Not employed
   7. Other: _______
9. Please specify what age group you primarily want to work with after receiving your highest degree.
   1. Birth to 3 years
   2. Preschool
   3. Elementary School-aged
   4. Middle School-aged
   5. High School-aged
   6. College/University-aged
   7. Adults
   8. Seniors
   9. Other
10. Please select all the clinical population you have studied in your coursework:
    1. Aphasia
    2. Apraxia of speech
    3. Autism
    4. Developmental language disorders
    5. Cleft lip and cleft palate
    6. Cognitive-communication disorders
    7. Dementia
    8. Dysarthria
    9. Dysphagia (adult)
    10. Dysphagia (pediatric)
    11. Fluency disorders
    12. Intellectual disabilities (e.g. Down Syndrome, Fragile X)
    13. Preschool language disorders
    14. Social communication disorder
    15. Severe disabilities
    16. Selective mutism
    17. Speech sound disorders: articulation and phonology
    18. Voice and voice disorders

**Supplementary Materials C**

Table 1

*Educational and Clinical Experience of SLP Sample*

|  | Graduate Student  (*n* = 16) | | Non-Graduate Student  (*n* = 61) | |  |  |  |  |  |
| --- | --- | --- | --- | --- | --- | --- | --- | --- | --- |
| Highest Degree Earned | |  |  | | |  | |  |  |
| Bachelor’s | | 43.8% | 4.9% | | |  |  |  |  |
| Master’s | | 56.3% | 85.2% | | |  |  |  |  |
| Doctorate (PhD or Clinical) | | -- | 9.9% | | |  |  |  |  |
|  | |  |  | | |  |  |  |  |
| Certified | | 43.8% | 98.36% | | |  |  |  |  |
| Primary Employment Facility | |  |  | | |  |  |  |  |
| Early Intervention | | 18.8% |  | 13.1% | |  | | |  |
| PreK | | 37.5% |  | 14.8% | |  | | |  |
| K-12 | | 37.5% |  | 24.6% | |  | | |  |
| College/University | | 25.0% |  | 8.2% | |  | | |  |
| Hospital | | 25.0% |  | 4.9% | |  | | |  |
| Residential Health | | -- |  | -- | |  | | |  |
| Nonresidential Health | | 12.5% |  | 9.8% | |  | | |  |
| Private Practice | | 31.3% |  | 29.5% | |  | | |  |
| Local/State/Federal Agency | | 56.3% |  | 39.3% | | |  | | |
| Research/Scientific Organization | | 50.0% |  | 16.4% | |  | | |  |
| Not Employed | | 1.0% |  | 6.6% | |  | | |  |
| Primary Age Group of Practice | |  |  |  | |  | | |  |
| Early Intervention | | 25.0%% |  | 44.3% | |  | | |  |
| PreK | | 18.8% |  | 65% | |  | | |  |
| Elementary School | | 31.3% |  | 67.2% | |  | | |  |
| Middle School | | -- |  | -- | |  | | |  |
| High School | | 12.5% |  | 36.1% | |  | | |  |
| College/University | | -- |  | 29.5% | |  | | |  |
| Adults | | -- |  | 19.7% | |  | | |  |
| Seniors | | -- |  | 8.2% | |  | | |  |
| Other | | 6.3% |  | 11.5% | |  | | |  |
|  | |  |  |  | |  | | |  |

*Note.* Percentage of the sample based on the highest degree earned, certification, primary employment facility, and primary age group of practice by graduate student status. For primary employment facility and age group of practice, participants could select more than one option. Therefore, percentages do not sum to 100%. Questionnaire can be found in Supplementary Materials B.

Table 2

*Mean (SD) Confidence Ratings for Each Word Learning Principle by Participants with Aligned vs. Different Intuitions*

|  | Aligned/Different Intuition on Word Learning Principle | |  |  |
| --- | --- | --- | --- | --- |
| Variable | Correct | Incorrect | t | p |
| Mutual Exclusivity | 2.67 (1.11) | 2.33 (1.22) | 0.79 | .44 |
| Whole Object Assumption | 3.28 (0.94) | 2.40 (1.07) | 2.47 | .03 |
| Taxonomic Bias | -- | -- | -- | -- |
| Shape Bias | 3.02 (0.99) | 2.58 (0.91) | 2.19 | .03 |
| Pointing During Learning | 3.86 (0.91) | 3.00 (--) | -- | -- |
| Looking to Visible Object | 3.03 (1.10) | 2.74 (1.13) | 1.13 | .26 |
| Overheard Speech | 2.77 (1.15) | 2.10 (0.74) | 1.01 | .30 |
| Looking to Non-Visible Object | 3.14 (0.89) | 2.94 (0.68) | 2.54 | .02 |
| Massed vs. Spaced Learning  CSWL | 2.88 (0.93) | 3.06 (0.93) | -0.97 | .34 |
|  | 2.71 (0.71) | 2.89 (1.08) | -0.98 | .33 |
| Context | 2.93 (0.95) | 3.11 (0.98) | -0.92 | .36 |

*Note.* Confidence ratings by alignment on word learning principle using a 1-5 Likert scale (1 = “Not confident at all”, 5 = “Extremely confident”;). A series of independent samples *t*-tests, Bonferroni-corrected for multiple comparisons (α = .004), assessed differences in mean confidence ratings between participants with aligned vs. different intuitions for each principle. Only one participant answered incorrectly on the principle testing the importance of pointing during learning. As a result, no standard deviation is reported for this principle.

Table 3

*Mean (SD) Interest Ratings for Each Word Learning Principle by Participants with Aligned vs. Different Intuitions*

|  | Aligned/Different Intuition on Word Learning Principle | |  |  |
| --- | --- | --- | --- | --- |
| Variable | Correct | Incorrect | t | p |
| Mutual Exclusivity | 3.06 (0.84) | 3.67 (0.87) | -2.02 | .07 |
| Whole Object Assumption | 3.03 (0.86) | 3.00 (0.82) | 0.13 | .90 |
| Taxonomic Bias | -- | -- | -- | -- |
| Shape Bias | 3.13 (0.90) | 3.11 (0.98) | 0.10 | .92 |
| Pointing During Learning | 3.03 (0.85) | 2.00 (--) | -- | -- |
| Looking to Visible Object | 2.81 (0.99) | 3.04 (0.89) | 0.30 | .77 |
| Overheard Speech | 2.83 (0.93) | 2.90 (1.20) | 0.19 | .85 |
| Looking to Non-Visible Object | 2.86 (0.82) | 2.81 (0.98) | -0.18 | .86 |
| Massed vs. Spaced Learning  CSWL | 3.04 (0.99) | 2.93 (0.77) | 0.58 | .56 |
|  | 2.86 (0.85) | 3.04 (0.89) | -0.96 | .34 |
| Context | 3.05 (0.76) | 2.82 (0.98) | 1.29 | .20 |

*Note.* Interest ratings by alignment on word learning principle using a 1-5 Likert scale (1 = “Not interested at all”, 5 = “Extremely interested”). A series of independent samples *t*-tests, Bonferroni-corrected for multiple comparisons (α = .004), assessed differences in mean confidence and interest ratings between participants with aligned vs. different intuitions for each principle. Only one participant answered incorrectly on the principle testing the importance of pointing during learning. As a result, no standard deviation is reported for this principle.

# Supplementary Materials D

**Exploratory Analyses**

**Age of Youngest Child.** As an exploratory analysis, we conducted a logistic regression with age of youngest child entered as a predictor for the sample from the general public/parents. We predicted that more recent experience with early language development may impact adults’ intuitions. Results revealed that age of the youngest child did not significantly predict responses on any of the word learning principles, *ps* > .99.

**Education Level.** To determine the role of educational level on intuitions, we conducted a logistic regression with education level entered as a predictor for the sample from the general public/non-parents and general public/parents. We predicted that educational level (1 = some college, college degree, or postgraduate degree, 0 = no some college, college, or postgraduate degree) may impact adults’ intuitions. Controlling for age and parenthood status, having some college, a college degree, or a postgraduate degree (*n* = 68) did not significantly predict responses on any of the word learning principles, *ps* > .99.

**Occupational Industry.** To determine the role of occupational experience on intuitions, we conducted a logistic regression with industry entered as a predictor for the sample from the general public/non-parents and general public/parents. We predicted that occupational experience in the field of education (i.e., “K-12 Education”, “College, University, and Adult education”, “Other Education Industry”) may impact adults’ intuitions. Results revealed that, controlling for age and parenthood status, experience in the field of education did not significantly predict responses on any of the word learning principles, *ps* > .99.

**SLP Clinical Experience.** We were interested in whether the age group a SLP works with in their clinical practice affected intuitions on the tested principles. We conducted a logistic regression with working in early intervention or with preschoolers dummy-coded (1 = working in early intervention or with preschoolers, 0 = not working in early intervention or with preschoolers) with SLP participants. Controlling for being a parent, SLPs who worked in early intervention or with preschoolers (*n* = 42) did not differ from SLPs who worked with other age groups (*n* = 31), except for in their judgments of massed vs. spaced learning: The relative chance of SLPs working with young children claiming spaced learning as better than massed learning was 30.93% of the relative chance of SLPs not working in this age group, *β* = -1.17, *SE* = .59, *z* = -1.98, *p* = .04. That is, SLPs who worked with young children were less likely to correctly identify spaced learning as better than massed learning, relative to SLPs who did not work with young children. We observed this similar trend with parents in the analyses in Experiment 1, suggesting that more in/formal experience with young children leads to intuitions that are less aligned with research for massed versus spaced learning.
